# Anti-Inflammatory Targets for the Treatment of Reperfusion Injury in Stroke

**DOI:** 10.3389/fneur.2017.00467

**Published:** 2017-09-07

**Authors:** Atsushi Mizuma, Midori A. Yenari

**Affiliations:** ^1^Department of Neurology, University of California, San Francisco and Veterans Affairs Medical Center, San Francisco, CA, United States

**Keywords:** reperfusion injury, postischemic inflammation, microglia, hyperglycemia, ischemic stroke, revascularization, tissue plasminogen activator, endovascular treatment

## Abstract

While the mainstay of acute stroke treatment includes revascularization via recombinant tissue plasminogen activator or mechanical thrombectomy, only a minority of stroke patients are eligible for treatment, as delayed treatment can lead to worsened outcome. This worsened outcome at the experimental level has been attributed to an entity known as reperfusion injury (R/I). R/I is occurred when revascularization is delayed after critical brain and vascular injury has occurred, so that when oxygenated blood is restored, ischemic damage is increased, rather than decreased. R/I can increase lesion size and also worsen blood barrier breakdown and lead to brain edema and hemorrhage. A major mechanism underlying R/I is that of poststroke inflammation. The poststroke immune response consists of the aberrant activation of glial cell, infiltration of peripheral leukocytes, and the release of damage-associated molecular pattern (DAMP) molecules elaborated by ischemic cells of the brain. Inflammatory mediators involved in this response include cytokines, chemokines, adhesion molecules, and several immune molecule effectors such as matrix metalloproteinases-9, inducible nitric oxide synthase, nitric oxide, and reactive oxygen species. Several experimental studies over the years have characterized these molecules and have shown that their inhibition improves neurological outcome. Yet, numerous clinical studies failed to demonstrate any positive outcomes in stroke patients. However, many of these clinical trials were carried out before the routine use of revascularization therapies. In this review, we cover mechanisms of inflammation involved in R/I, therapeutic targets, and relevant experimental and clinical studies, which might stimulate renewed interest in designing clinical trials to specifically target R/I. We propose that by targeting anti-inflammatory targets in R/I as a combined therapy, it may be possible to further improve outcomes from pharmacological thrombolysis or mechanical thrombectomy.

## Introduction

Treatment of acute ischemic stroke has largely been limited to strategies to restore blood flow. Pharmacological recanalization, particularly tissue plasminogen activator (tPA) has been the mainstay for acute treatment for the past 20 years (1, 2), but in recent years, several studies have shown that mechanical embolectomy is also effective (3). However, the short time frame for safe intervention is still limited even considering recent studies that suggest additional criteria for treating “wake up stroke” (4) and longer time windows of up to 24 h for embolectomy (5). It is still estimated that less than 10% of all acute stroke patients benefit from reperfusion strategies. One of the reasons for such a short time window is that intervention beyond this time window actually increases risk and leads to worsened outcome (6). If these recanalization therapies are applied too late, there is an increased risk of cerebral hemorrhage, which can sometimes prove fatal (7). The mechanism of cerebral hemorrhage complicating ischemic stroke is a consequence of a phenomenon known as “reperfusion injury” (R/I) (8) to which inflammation is a major contributing cause.

**Figure 1:**
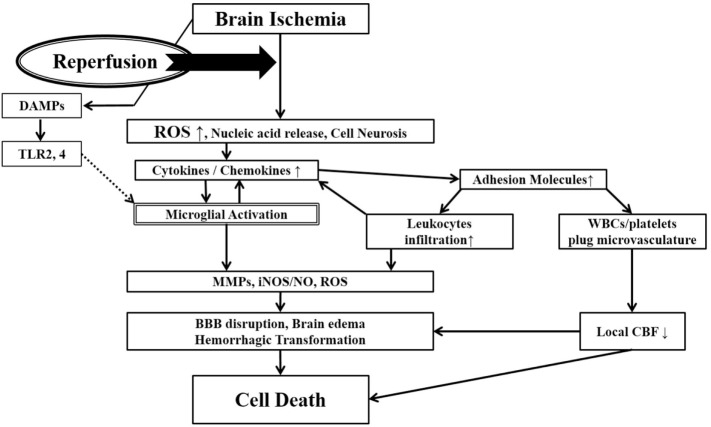
Ischemia-induced inflammation in association with reperfusion injury. Once brain ischemia occurs, oxygen and glucose supplies are reduced. If ischemia occurs for more than a certain time period (likely a few hours, but the precise duration is not well established) and blood flow is restored (reperfusion), worsened injury can paradoxically occur to the brain. This is often referred to as reperfusion injury. A major component of reperfusion injury involves subsequent inflammatory reactions induced through various mechanisms. Reperfusion leads to the introduction of ROS from oxygenated blood and can stimulate an immune response in the ischemic brain. Necrotic, ischemia-injured cells lyse and release their contents into the extracelluar space which can act as ligands for various immune receptors. Among these include nucleic acids which are one of many described damage-associated molecular pattern (DAMPs, see text for details). DAMPs can then bind TLRs and stimulate several inflammatory responses (microglial activation, overexpression of proinflammatory cytokines, chemokines) which lead to worsened brain injury. Inflammatory signaling also causes immune cells to generate more effector molecules such as ROS and iNOS/NO. In the periphery, cytokines and adhesion molecules can attract circulating immune cells to the ischemic brain where they infiltrate the damaged tissue and further amplify ischemic injury. Some circulating immune cells and platelets can also plug the microvasculature of the ischemic brain and cause secondary reductions in local CBF. In addition to brain cells, these inflammatory reactions can also cause damage to brain endothelia causing BBB disruption, edema and hemorrhagic transformation. Thus, the restoration of CBF can cause more extensive brain tissue damage. This vicious cycle is often called reperfusion injury. ROS, reactive oxygen species; DAMPs, damage-associated molecular patterns; TLR, toll-like receptor; MMPs, matrix metalloproteinases; iNOS, inducible nitric oxide synthase; NO, nitric oxide; BBB, blood–brain barrier; CBF, cerebral blood flow.

While the restoration of cerebral blood flow (CBF) is a major goal of acute stroke treatment, it can also lead to more extensive brain tissue damage in the adjacent penumbral territory (9). If recanalization is carried out after a critical time window, the sudden restoration of oxygenated blood into ischemically compromised brain tissue may overwhelm already compromised endogenous antioxidant systems and damaged vascular endothelia leading to brain edema and extravasation of blood cells. The generation of reactive oxygen species (ROS) from compromised mitochondria not only leads to direct cellular damage but also can trigger the activation of both the peripheral (leukocytes) and brain resident (microglia) immune pathways, which in turn, elaborate various damaging immune mediators and effectors including more ROS. This vicious cycle in acute ischemic stroke is referred to as cerebral R/I (Figure 1) (10, 11).

The evidence for R/I was previously demonstrated using experimental stroke models. A few groups have reported that ischemic injury is greater in animals where reperfusion occurs [temporary middle cerebral artery occlusion (tMCAO) for 2–3 h] compared to animals where there is no reperfusion (pMCAO) (12, 13). In a series of experiments where the duration of MCAO was varied and compared to pMCAO, tMCAO for less than 2 h led to smaller infarct sizes compared to pMCAO (14). Occlusion durations of more than 2 h led to paradoxically larger infarct volumes. However, direct evidence for R/I is less clear at the clinical level. While a rare “hyperperfusion syndrome” of accelerated brain edema and transient clinical worsening following abrupt revascularization has been described (15), it is not clear whether this results in permanent worsened outcome. Further, the neurotoxicity of tPA has been shown in previous studies, where endogenous tPA may directly contribute to worsened outcome (16). Further, it is quite clear that revascularization after certain time windows can worsen outcomes compared to no intervention (6) and could be said to represent R/I in humans. Hence, targeting aspects of R/I might suggest an opportunity to synergistically improve neurological outcome for thrombolysis and/or mechanical embolectomy.

While the concept of R/I as a therapeutic target surrounding revascularization, the efficacy of treatment in experimental reperfusion models does not necessarily predict the results of clinical trials. A PubMed search for experimental studies covering the terms “reperfusion injury, cerebral ischemia, and inflammation” revealed that 888 studies have been performed using the tMCAO model. However, only one agent (edaravone) has actually been transition to the clinical level in Japan. Experimental reperfusion models do not fully replicate what happens in clinical stroke. Hence some reports argue that experimental reperfusion models were inappropriate for clinical translation (17). Regardless, the timing of treatment is different for each clinical case. These factors are major problems that cannot be avoided. However, some novel mechanisms associated with R/I have been established in over the years by studying experimental models and may suggest therapeutic targets which could be studied at the clinical level in this new era of recanalization.

In this review, we will focus on the mechanism of R/I in acute ischemic stroke and reconsider its treatment, with a focus on proinflammatory targets, including some already in use at the clinical level.

## Inflammation in Stroke

The poststroke inflammatory response consists of aberrant activation of glial cells, particularly microglia, and the infiltration of peripheral leukocytes, following exposure to molecules containing damage-associated molecular patterns (DAMPs) released by ischemic brain cells (Figure 1) (18–22). DAMPs studied in brain ischemia include HMGB-1, purines, nucleotides such as ATP and UDP, and nucleic acid fragments (15). DAMPs stimulate immune cell production of cytokines/chemokines, adhesion molecules, and several immune molecule effectors such as matrix metalloproteinases-9 (MMP-9), inducible nitric oxide synthase (iNOS), nitric oxide (NO), ROS, and nicotinamide adenine dinucleotide phosphate (NADPH) oxidase, all of which lead to exacerbation of cerebral ischemic injury (18, 20, 21).

### Microglial/Macrophage Activation

Microglia are the brain’s resident immune cell (23) and are involved in the modulation of the brain’s inflammatory response. Circulating monocytes are also recruited from bloodstream and enter brain as macrophage and associated with inflammation (24). Once injury occurs, microglia and macrophage are activated and release proinflammatory molecules such as cytokines [e.g., tumor necrosis factor-α (TNF-α), interleukin-1β [IL-1β], IL-6, and interferon-γ (IFN-γ)], chemokines, NO, ROS, MMP-9, glutamate, and ATP (25–32). Microglia are immediately activated following ischemia, elaborating various immune molecules which set the stage for the recruitment of circulating leukocytes (27). Activated microglia affects to neuroinflammation as macrophage-like cells (33). Macrophages also enter the brain from the circulation as early as 24 h after stroke onset. Their numbers in ischemic lesions peak within 3–7 days (34) but remain within the lesion for weeks to even months (35–39). Phagocytic microglia or infiltrated circulating macrophages are thought to remove necrotic debris and other potentially damaging substances, paving the way for recovery and repair (40).

Interestingly, while activated microglia/macrophage have been thought to worsen damage in stroke, they also appear to contribute to their protection (35, 41–44). Recent work has now shown that activated microglia/macrophage can be classified into two broad subpopulations—M1 and M2, with the former being proinflammatory and the latter being anti-inflammatory (33, 45). Acutely, microglia appear to polarize predominantly to a M1 phenotype and produce cytokines, ROS, and reactive nitrogen species (45). As such, iNOS is the major NOS in immune cells that leads to NO generation, and is often used as a M1 marker (46). The M1 phenotype is mainly associated with proinflammatory signaling (47). In contrast, the M2 phenotype contributes to anti-inflammatory responses as well as recovery and repair (45). Cytokines associated with the M2 phenotype include IL-4, IL-10, IL-13, IGF-1, and transforming growth factor (TGF)-β (33, 45, 48). These cytokines are also important for protection from inflammation (33, 48–53). Arginase 1, which competes with iNOS and downregulates NO production, is often used as a marker for M2 activation (54).

Several innate immune receptors have been studied in relation to activated microglia and the inflammatory cascade. These receptors have in common their ability to activate when stimulated by DAMPs. Purinergic receptors, especially P2X_7_ and P2Y_12_, were previously reported to modulate microglial activation and mediate neurotoxicity (55). Similarly, pharmacologic inhibition of P2X_7_ and P2Y_12_ reduced brain injury in experimental stroke models (55). Other innate immune receptors studied in stroke include toll-like receptors (TLRs), transient receptor potential melastatin 2, CD36 scavenger receptors, and receptor for advanced glycosylation endproducts (RAGE) (56–60).

### Astrocyte

Astrocytes also play an important role as the source of various inflammatory molecules (61). Activated astrocytes are sometimes referred to as “reactive gliosis” after ischemia and have been observed 4–24 h after ischemia onset, peaking at around 4 days. This state is accompanied by astrocytic upregulation of glial fibrillary acidic protein. The resulting glial scar is a consequence of reactive cellular processes that consists of activated astrocytes and has an important role which leads to both benefit and harm (62). While this glial scar acts as a barrier surrounding the ischemic lesion, it may also prevent axonal regrowth (63). Further, activated astrocytes are directly associated with inflammatory responses after ischemia through upregulation of major histocompatibility complex and other inflammatory molecules such as cytokines, chemokines and iNOS (61). These molecules can exacerbate ischemic injury. In contrast, activated astrocytes are also capable of developing Th2 (anti-inflammatory) responses, although this mechanism has not been reported in ischemia (64). Further, some astrocyte derived immune molecules have been shown to have contrasting effects in a model of spinal cord injury (65). Thus, the modulation of activated astrocytes may be critical in influencing the role of inflammation in brain ischemia, although this area of investigation is still quite new.

### Leukocyte Infiltration

There are very few peripheral leukocytes in uninjured brain, but once an ischemic stroke occurs, endothelial cells within the cerebral microvasculature are activated and leukocyte infiltration follows a few hours later (41, 66, 67). This immune cell activation and infiltration appears to be more robust following tMCAO compared to pMCAO (68). Once infiltrated, leukocytes contribute to the worsening of brain damage through similar mechanisms as activated microglia (18, 35). In addition, circulating leukocytes also lead to platelet aggregation through activation of the arachidonic acid cascade (i.e., leukotrienes, thromboxane, and prostaglandins) (69). This aggregation promotes a prothrombotic state and induces a “no-reflow” state which can also further compromise ischemic tissue (61, 70). Leukocyte–platelet complexes are formed which causes further damage by plugging the microvasculature. Thus, circulating leukocytes worsen ischemic injury through direct infiltration into the ischemic brain and by obstructing *in situ* blood flow (61).

#### Neutrophils

Neutrophils are the first population of leukocytes to appear in the acute ischemic brain (71, 72). While they function as any other effector immune cell, their significance has been debated. Several studies have shown that inhibiting their ability to infiltrate ischemic brain led to smaller lesion sizes (61). However, numbers of neutrophils in the ischemic brain do not appear to predict stroke severity (73–75). Further, this strategy has not been shown to improve outcomes in stroke patients (76). In addition, Enzmann et al. (75) argued that neutrophil infiltration was not present in infarcts at an early stage after ischemia. In this study, platelet aggregation, sites of increased vessel permeability, and sites of enhanced expression of endothelial adhesion molecules were also correlated with neutrophils infiltration. Hence, the role of neutrophils in stroke is still unclear, particularly since clinical studies to inhibit their infiltration were conflicting.

#### Lymphocytes

Activated T lymphocytes also appear in ischemic brain tissue as early as 24 h after reperfusion, but later than neutrophils and before macrophages (77). They act as a modulators of leukocyte and platelet adhesion (78). T lymphocytes also produce inflammatory cytokines such as IL-17 and IFN-γ. CD4^+^helper, CD8^+^cytotoxic, and γδT cells have been shown to play harmful roles in experimental stroke (78, 79). Yilmaz et al. (79) reported that the deficiency of CD4^+^helper/CD8^+^cytotoxic T cells led to smaller infarct sizes, lower number of adherent leukocytes and lymphocytes, and better neurological outcomes following tMCAO in mice. γδT lymphocytes mainly produce IL-17, and IL-17 seems to play an important role in the delayed phase of R/I (78). Hence, they may be important therapeutic targets. In fact, high levels of IL-17 have been documented in human ischemic lesions (80). IL-23 also appears to mediate IL-17. Shichita et al. (78) showed infarct volume reduction and improvement of neurological functions in IL-17/IL-23 KO mice. However, while the harmful role of T lymphocytes in brain ischemia was demonstrated in a tMCAO model and R/I, the same has not been shown in pMCAO for unclear reasons (81). Further, T regulatory cells (T_regs_) seem to play an anti-inflammatory and neuroprotective role in ischemic stroke. T_regs_ interact with other cells to release anti-inflammatory cytokines (IL-10 and TGF-β) (24). Liesz et al. (82) reported that the depletion of T_regs_ increased infarct volume and worsened of neurological function. Further, anti-CD28 antibody (CD28SA) was shown to reduce infarct volume and improve neurological deficit by increasing numbers of T_regs_ (83). In contrast, Kleinschnitz et al. argued that T_regs_ could promote IS *via* cerebral microvasculature dysfunction, as T_regs_ depletion was neuroprotective (84). Further, CD28SA contributed to enhanced T_regs_ effects by promoting thromboinflammation T_regs_ have also been reported to inhibit t-PA induced hemorrhagic transformation (HTf) through regulation of MMP-9 and suggests T_regs_ as a potentially important target in preventing this complication in the setting of recombinant tPA use (85). Clearly, T_regs_ in acute IS is complex and more investigation is needed. B lymphocytes also contribute to the reduction of inflammation and neurological deficits through IL-10 release (86).

In clinical studies, the relationship between lymphocyte numbers and poststroke pathology is not clear. Nadareishvili et al. (87) reported that high serum lymphocyte counts increased the risk of stroke recurrence and mortality. In contrast, Kim et al. (88) reported that lower lymphocyte counts were associated with poor early neurologic improvement and worse long-term functional outcome.

### Cytokines/Chemokines

Several cytokines and chemokines are released following ischemic brain injury (19, 20). They have been detected in blood, cerebrospinal fluid, and infarcted brain regions in both acute ischemic stroke patients and experimental animals (20). In microglia, distinct molecules are expressed, depending on the cell’s phenotype (23, 33). M1 microglia generate proinflammatory cytokines, which contribute to the worsening of brain injury (45). The most extensively studied of the proinflammatory cytokines include IL-1, IL-6, and TNF-α (89). Cytokines from M2 phenotype microglia appear to contribute a protective or beneficial effect, and are thought to inhibit inflammation and promote tissue repair (33, 48–53). Representative anti-inflammatory cytokines studied in stroke are IL-4, IL-10, IL-23, and TGF-β1 (33, 45, 48). Several chemokines have been studied in stroke models and appear to play a variety of roles in cell migration and attraction to sites of damage.

#### Proinflammatory Cytokines

Interleukin-1 is consists of two isoforms IL-1α and IL-1β, which are major contributors of acute inflammation in ischemic stroke and have been studied as therapeutic targets (90). IL-1 also indirectly affects brain injury through initiating astrogliosis, releasing toxic substances [chemokines, vascular cell adhesion molecule (VCAM)-1, and intercellular adhesion molecule (ICAM)-1], and activating metalloprotenase (MMP)-9 (91–93). Both isoforms increase in the first hours of acute ischemic stroke and bind to their corresponding receptors (IL-1R1 and IL-1R2) (24, 61, 90). Previous studies showed that IL-1β contributed more to ischemic pathology rather than IL-1α (93). IL-1β mRNA and protein are detected within 1–6 h after ischemia with a biphasic pattern of expression that includes a secondary peak 6–24 h later (94). Administration of IL-1β led to the enlargement of infarct size (95), whereas the lack of IL-1β was led to smaller infarct size (93). Similarly, studies have explored the feasibility of administering its endogenous inhibitor, IL-1ra, which seems to improve outcomes in experimental models.

Interleukin-6 has also been studied in ischemic stroke, but its role has not been clarified. The expression and increase of IL-6 is observed as early as 3 h, peaking at 12 h, and remains elevated at 24 h after onset of brain ischemia (96). IL-6 has been detected in plasma and cerebrospinal fluid of stroke patients, and IL-6 immunoreactivity has been detected in microglia and cortical neurons of ischemic stroke models (96). In patients, IL-6R polymorphisms are also related to poor neurological outcomes (97), although IL-6 polymorphisms did not show an increased risk of ischemic stroke (98). To add to the conflicting observations, IL-6-deficient mice and mice treated with an IL-6 receptor antagonist mice did not have improved outcomes compared to untreated mice (99, 100).

Tumor necrosis factor-α has also been detected in ischemic brain (96) and appears largely in microglia as early as 30 min after experimental stroke (96). This expression has been observed in a variety of other brain cells including neurons and astrocytes. Interestingly, TNF-α has been reported to be both neurotoxic and neuroprotective in the brain. Yang et al. (101) reported that administration of neutralizing antibodies to TNF-α showed a protective effect against ischemic injury in tMCAO model. In contrast, Bruce et al. (102) reported that ischemic brain injury was increased in mice genetically deficient in the TNF receptor. Reasons for this discrepancy could be explained by its two different surface receptors (TNFR1 and TNFR2) (103). TNF-α exists in transmembrane and soluble forms. Soluble TNF-α binds to TNFR1 leading to neurotoxic effects, whereas membrane bound TNF-α binds to TNFR2 leading to neuroprotective effects (104). However, Pradillo et al. (105) reported that TNFR1 signaling pathways led to resistance to ischemic injury through upregulation of TNF-α convertase enzyme and TNFR1. To confuse matters more, it also appears that the effects of TNF-α induced in striatum are neurodegenerative, whereas that in the hippocampus is neuroprotective (106).

T cell-releasing proinflammatory cytokines are also important in the development of ischemic stroke (77). IFN-γ, IL-17, and IL-23 have been studied recently as therapeutic targets (78). IL-17 induces TNF-α, IL-1β, and MMP-9 in macrophages and expression and IL-23 induces the expression of IL-17 (107, 108). As such, IL-17 and IL-23 both contribute to the worsening of ischemic injury.

#### Anti-Inflammatory Cytokines

Interleukin-10 is an anti-inflammatory cytokine mainly produced by microglia, macrophages, and astrocytes, and has a neuroprotective effect in ischemic injury (33, 61). IL-10 acts by inhibiting proinflammatory cytokines, such as IL-1β, TNF-α, and IFN-γ, and suppresses the expression and activation of cytokine receptors (61, 82). Overexpression or administration of IL-10 has been reported to reduce brain injury and improve neurological outcomes in experimental stroke. Ooboshi et al. (109) reported that postischemic gene transfer of IL-10 into the lateral ventricle reduced brain infarct size. At the clinical level, lower IL-10 levels increased stroke risk (110). T_regs_ also produce IL-10 (24, 82) and Li et al. (111) reported that administration of T_regs_ reduced immune cell infiltration, brain inflammation, and prevented blood–brain barrier (BBB) disruption.

Transforming growth factor-β1 is also produced by microglia, macrophages, and astrocytes after ischemia and has also been shown to protect from ischemic injury (112). Like IL-10, overexpression or administration of TGF-β1 reduced ischemic lesion size and improved neurological outcomes in experimental stroke model (113, 114).

Interleukin-4 is another widely studied anti-inflammatory cytokine produced by activated Th2 cells, eosinophils, basophils, and mast cells (115). IL-4 signaling is mediated through IL-4 receptor α-chain (IL-4Rα) on neutrophils, T lymphocytes, astrocytes, oligodendrocytes, and endothelial cells (116, 117). After binding, this complex dimerizes with the common γ-chain or IL-13Rα1 which is a closely related cytokine receptor (118). This signaling contributes to potent anti-inflammatory responses in ischemic brain which include upregulating Arg1, Ym1, found in inflammatory zone 1 (FIZZ1), CD206 (mannose receptor), chemokine ligand 22 (CCL22), and CD163, which are also M2 specific markers (117). IL-4 inhibits the release of proinflammatory cytokine genes such as IL-1β, TNF-α, IL-6, and chemokines, and the activation of macrophage mannose receptor (119). IL-4 levels in brain are generally very low, but increase several hours after stroke onset (116). Xiong et al. (51) reported that IL-4 deficiency exacerbated brain injury and worsened neurological outcomes in tMCAO. Further, IL-4 appears to have long-term neuroprotective effects after experimental stroke (116). However, it bears mentioning that other studies reported that IL-4 was associated with proinflammatory responses (120). Pretreatment of monocytes and macrophages with IL-4 has also been shown to enhance proinflammatory cytokine gene expression such as TNF-α, IL1-α, and macrophage-inflammatory protein-2 (121).

#### Chemokines

Chemokines mediate chemotaxis, including leukocyte migration (122). They also participate in the pathogenesis of cerebral ischemia through their involvement in leukocyte infiltration, ROS release, and BBB disruption (24, 61). Based on their structures, chemokines are divided into four subgroups; CXC, CC, C, and CX_3_C (123). Corresponding receptors include CXCR (1–5), CCR (1–9), XCR1, and CX_3_CR1 (61). Both chemokines and their receptors have been explored as potential therapeutic targets (24, 123).

Stromal cell-derived factor (SDF)-1 (CXCL12) is a CXC chemokine (124). It is produced by bone marrow stromal cells and acts as a strong chemoattractant for CD34+ cells, which express SDF-1α receptor (CXCR4) (124). SDF-1 α/CXCR4 signaling plays an important role in ischemia-induced inflammation *via* activation and migration of leukocytes (125). Ruscher et al. (126) reported that the inhibition of CXCL12 reduced infiltration of immune cells and Huang et al. (127) also confirmed that CXCR4 antagonism led to neurological recovery, suppression of proinflammatory cell infiltration, reduction of infarct area, and BBB disruption in tMCAO.

Fractalkine (CX_3_CL1) is a CX_3_C chemokine that is mainly expressed on neurons and acts through CX_3_CR1 on microglia (128). Fractalkine bound to CX_3_CR1 leads to expression of proinflammatory cytokines, ROS production, BBB disruption, and leukocyte infiltration (24, 128). However, the role of CXC_3_CL1/CXC_3_R1 signaling is still controversial. While Soriano et al. (129) reported that fractalkine deficiency reduced infarct volume and mortality in tMCAO, Cipriani et al. (130) showed that administration of CX_3_CL1 reduced ischemic injury. Reasons for these discrepancies are unclear.

Of the CC chemokines, monocyte chemoattractant protein (MCP)-1 (CCL2) and macrophage inflammatory protein-1α (CCL-3) have been the most studied and participate in leukocyte infiltration (24, 131). CCL2 and CCL3 are mainly expressed on astrocytes, microglia, and monocytes, and CC chemokine inhibition reduced brain injury (132). CC chemokines also lead to expression and secretion of Regulated upon Activation, Normal T-cell Expressed, and Secreted, also known as C-C motif ligand 5 (CCL5) on T cells (123). CCL5 mediates the migration and adhesion of leukocytes through three different types of G protein-coupled receptors (CCR1, CCR3, and CCR5). Victoria et al. (133) confirmed that CCR5 deficiency led to improvement of neurological deficits and reduction of infarct volume against R/I. CCL5 also forms a heterodimer with CXCL4 and constitutes a CXC subgroup. CXCL4-CCL5 enhances the effect of CCL5, which contributes to ischemia-induced inflammation and brain injury, against which a CXCL4/CCL5 inhibitor protects (134).

### Cell Adhesion Molecules

Cell adhesion molecules (CAMs) are involved in the trafficking and recruitment of leukocytes to activated ischemic endothelia in stroke. They contribute to the inflammatory response and worsen ischemic brain injury (135). CAMs are divided into three sub-groups; the immunoglobulin superfamily [ICAM-1, 2, VCAM-1, platelet-endothelial cell adhesion molecule-1 (PECAM-1), the mucosal adhesion (MAdCAM-1), and activated leukocyte cell adhesion molecule], selectins [P-selectin (CD62P), E-selectin (CD62E), and L-selectin (CD62L)], and integrins (CD11, CD18, CD29, and CD49) (20). Following stroke, high levels of CAMs are expressed on cerebral endothelium, which lead to the recruitment and rolling of leukocytes and platelets in the cerebral microvasculature. When these rolling leukocytes approach activated endothelium, they adhere firmly to endothelial cells, after which transendothelial migration occurs leading to infiltration of ischemic brain (20, 61, 66). These processes are also thought to be responsible for the “no flow phenomenon” of endothelial dysfunction.

Selectins as therapeutic targets have also been studied in the past. Goussev et al. (136) showed that administration of anti-P-selectin antibody reduced ischemic injury and HTf in a stroke model. Huang et al. (137) also reported the same for antibodies against anti-E-selectin. In contrast, the role of L-selectin, which presents on leukocytes and mediates leukocyte transmigration, is unclear. When it encounters activated endothelium, it is shed from the cell surface. However, it may not influence ischemic and I/R injury, as studies to inhibit it have not been shown to improve outcome or influence leukocyte infiltration in stroke models (61).

Integrins are distributed on the cell surface and contribute to cell–extracellular matrix (ECM) adhesion (138). They exist as heterodimers which consist of α and β subunits. The migration of leukocytes is regulated by a specific integrin, which associates with a common β_2_ chain (CD18). When leukocytes adhesion occurs, integrin CD18 binds to ICAM-1. Several α chains [e.g., CD11a (LFA-1) and CD11b (Mac-1)] combine with CD18 prior to adhesion to ICAM-1. Both Mac-1 deficiency and antibody inhibition of this integrin led to decreased stroke size and numbers of infiltrated neutrophils (61). In addition to leukocytes, monocytes and lymphocytes express other integrins composed of the β_1_ (CD29) and β_7_ (CD103) subunits. These subunits combine with the α_4_ integrin (CD49d) and interact with VCAM-1, MAdCAM-1, and the subendothelial matrix (61, 139).

Immunoglobulin superfamily members are also cell surface glycoproteins, which are expressed on endothelial cells, leukocytes, platelets, fibroblasts, and epithelial cell (20, 61). ICAM-1 (CD54) exists constitutively at low levels, and it is upregulated by cytokines following ischemia (20). Kitagawa et al. (140) and Zhang et al. (141) reported that ICAM-1 deficiency or anti-ICAM-1 therapy reduced ischemic injury and leukocyte adhesion. VCAM-1 (CD106) is upregulated by proinflammatory cytokines TNF-α and IL-1. Although PECAM-1 (CD31) is not upregulated by cytokines, it plays an important role in endothelial cell adhesion and leukocytes transmigration (142). Liesz et al. (143) also found that VCAM-1 deficiency reduced T lymphocyte infiltration into the brain and reduced infarct volume. Studies of CD49d inhibition in two different ischemic stroke models (tMCAO and pMCAO) showed that infarct volume was reduced and leukocyte infiltration was suppressed (144).

However, the efficacy of antiadhesion molecule strategies has not been established at the clinical level. The Enlimomab trial, which evaluated the efficacy of an ICAM-1 antibody in acute IS patients, showed that was antibody-treated patients actually had worsening of neurological function, increased mortality and fever compared to the placebo group. The HALT and Acute Stroke Therapy by Inhibition of Neutrophils (ASTIN) studies, which examined strategies to target the CD11/CD18 leukointegrin were both negative, and were stopped early due to lack of efficacy (145).

### Immune Molecule Effectors

#### Matrix Metalloproteinase

Matrix metalloproteinases are a family of zinc-binding endopeptidases responsible for the ECM turnover and digestion of proteins (146). A variety of subtypes are classified based on structural similarity; collagenases, gelatinases, metalloelastases, stromelysins, membrane-type MMPs, and others (147). These proteases normally participate in wound healing, bone remodeling, angiogenesis and other physiological processes (61). However, inflammatory stimuli can lead to the activation of MMPs from their inactive proform to their cleaved active form through signaling *via* several proinflammatory cytokines (IL-1β) and chemokines (CCL-2, 7, 8, and 13) (148, 149). In stroke, MMPs have also been shown to participate in R/I. Activated MMP-2 and MMP-9 are known to contribute to the inflammatory response in acute ischemic stroke (147, 150). Microglia are a major source of MMPs (61) and activated MMPs have been detected within a few hours after ischemia onset and detected even 5 days later (61, 147). Activated MMP-2 and MMP-9 lead to the digestion of the endothelial basal lamina, including the degradation of tight junction proteins. This leads BBB disruption and secondary injury, causing brain edema and hemorrhagic transformation (151). tPA, which is present endogenously, also contributes to BBB and ECM disruption. Through its ability to activate plasmin, tPA indirectly activates MMPs, especially MMP-9, which is expressed by immune cells (151). In the setting of treatment with recombinant tPA (rt-PA), this could result in more severe BBB disruption and hemorrhage, both feared complications of pharmacological thrombolysis. Hence, MMP inhibition may be a particularly important means of reducing I/R injury.

Matrix metalloproteinase-9-deficient mice had smaller infarcts following tMCAO (152), and chimeric studies showed that MMP-9 expressed particularly by leukocytes contributed to worsened stroke outcome (153). Clinical studies showed that serum MMP-9 levels correlated with stroke severity, with MMP-9 mRNA as a predictor of poor outcome (154).

Matrix metalloproteinase-2 has similar properties as MMP-9; however, its roles in ischemic stroke are still unclear. Previous reports showed that MMP-2 deficiency in experimental stroke and serum MMP-2 levels in stroke patients did not affect ischemic lesion size or stroke severity, respectively (155). Yet, MMP-2 activity increases in the later phase of ischemic stroke (147) and basal MMP-2 levels were higher in patients with stable or recovering symptoms (156). MMP-2 elevations, at least in the acute phase of ischemic stroke, might also be associated with better clinical outcomes.

#### Inducible Nitric Oxide Synthase/Nitric oxide

Nitric oxide is synthesized from l-arginine and oxygen through nitric oxide synthase (NOS) (157). Three NOS isoforms are known: endothelial NOS (eNOS), neuronal NOS (nNOS), and iNOS (158). iNOS is increased in immune cells including leukocytes, microglia, macrophage, and possibly astrocytes in response to proinflammatory stimuli, while eNOS and nNOS exist in the vascular endothelium and neurons, respectively (159). Damaging effects of NO derived from iNOS have been shown in experimental stroke. Iadecola et al. (160) and Zhao et al. (161) reported that inhibition or deficiency of iNOS reduced infarct size and improved neurological outcomes. High serum levels of iNOS have also correlated with a worse outcome (162).

#### ROS/NADPH Oxidase

While ROS are generated from a variety of sources following stroke, immune cells generate ROS as part of their microbicidal functions. Several pro-oxidant enzymes exist within immune cells, but substantial work pertaining to stroke has centered around NADPH oxidase (NOX) (163, 164). NOX is a multicomponent enzyme consisting of six subunits within the plasma membrane subunits (gp91^phox^, gp22^phox^) and cytoplasm (gp47^phox^, gp67^phox^, gp40^phox^ and Rho family GTPases) (163). After activation, NOX is phosphorylated, and the cytoplasmic subunits translocate to the membrane and form a complete complex on the cell surface. When this occurs, superoxide is generated from oxygen by electron transfer from NADPH (163, 165). Different isoforms of NOX have been identified, with NOX1, 2, and 4 being described in ischemic injury (166–168). In experimental stroke models, deficiency of both gp91^phox^ and gp47^phox^ NOX subunits reduced lesion size, immune cell activation, and superoxide generation, as did pharmacological inhibition with apocynin (169). NOX4 was also shown to be a relevant therapeutic target, as its inhibition or deletion led to protection (170). These investigators also showed that NOX4 was a major source of oxidative stress.

### Transcriptional Regulation of Inflammation

Several transcription factors have been identified as regulators of the ischemic inflammatory response and also represent potential targets for intervention.

#### Nuclear Factor κB (NF-κB)

Nuclear factor-κB is a major transcription factor which contributes the inflammatory response, and has been documented in brain ischemia models (171). NF-κB exists as heterodimer composed of p65 (RelA) and p50 in the cytosol and bound by its inhibitor protein IkB. In the presence of an activating stimulus, IkB’s kinase phosphorylates IkB, freeing NF-κB to translocate to the nucleus where it binds its consensus sequences to upregulate many proinflammatory genes. Since NF-κB also upregulates genes involved in cell growth, its role is as a therapeutic target is still unclear and data are conflicting. On the one hand, deficiency or pharmacological inhibition of NF-κB was protective in some experimental stroke models (172, 173), but studies using a different inhibitor (diethyldithiocarbamate) found that this led to worsened outcome. Reasons for these discrepancies have been explained by the type of inhibitor used or whether NF-κB might be protective in some cell population, but damaging in others. Thus, treatment strategies specific targeting of NF-κB to differentially inhibit proinflammatory while upregulating prosurvival genes may be necessary.

#### Mitogen-Activated Protein Kinase (MAPK)

Mitogen-activated protein kinase has also been studied in ischemia-induced inflammation (174). It is involved in the regulation of inflammatory gene expression and apoptosis (175). Its three interlinked MAPK signaling pathways have been assessed in experimental stroke models; p38 MAPKs, stress-activated protein kinases/c-Jun N-terminal kinases (SAPK/JNK), and extracellular signal-regulated kinases (ERKs) (61). In particular, p38 MAPK activation has been documented in the production and activation of several inflammatory molecules in microglia, astrocytes, and neurons (175, 176). p38 MARK activation occurs *via* several kinases: MLK3, MKK3/MKK6, and MKK4. NO regulates the activity of p38 MARK through phosphorylation and S-nitrosylation. Inhibition of p38 MARK activation by SB239063 has been shown to protective against ischemic brain injury *via* suppression of proinflammatory cytokine/chemokine expression and synaptic dysfunction (177).

### Complement System

Complement system also plays crucial roles in association with IS and R/I and consists of secreted and membrane-bound proteins (178). When this system is activated, three major pathways are involved in its activation: the classical pathway (CP), the alternative pathway (AP), and the lectin pathway (LP). Once ischemia occurs, the complement system is activated *via* both of CP and LP (179). This complement cascade contributes to microglial activation, leukocyte adhesion and recruitment, promotion of phagocytic system, and direct cellular lysis (178–181). Several studies have been performed using experimental stroke models. Especially in the LP, mannose-binding lectin (MBL), which binds with the natural self-reactive IgM antibody and a LP-specific protease called mannan-binding lectin-associated serine proteases (MASPs)-2 have been recognized as key players. Antibody-deficient (Rag1^−/−^) or MBL-deficient mice exposed to experimental stroke were found to have reduced brain injury (182, 183). MASPs-2 antibody treatment also reduced infarct volume and improved neurological deficits in a similar model (179). In association with C3 cleavage, inhibition of complement receptor 2 which binds to C3d, led to the reduction of IgM and complement deposition, P-selectin, and neutrophil infiltration in experimental stroke (178).

### Damage-Associated Molecular Patterns

Recent work has begun to identify factors which trigger the inflammatory response in stroke and related insults. Like pathogen-associated molecular patterns (PAMPS), DAMPS include several molecules elaborated by injured cells which are able to bind any number of immune receptors. Some which have been identified in stroke and related brain injury models include high mobility group box-1, heat shock proteins, peroxiredoxin, nucleic acids, purines, and β-amyloid (61, 184). Neurons appear to be an early source of DAMPs in stroke. The complement system and several immune receptors recognize DAMPs and induce inflammation following IS and in association with R/I. Targets of DAMPs include several innate immune receptors, and those most studied in cerebral ischemic include the TLRs and purinergic receptors.

Toll-like receptors are present on the cell surface and act as a sensor of the innate immune response. TLR2 and TLR4 have been the most studied of these in stroke models (185). Following ischemic insults, brain cells elaborate DAMPs which then stimulate TLR2 and TLR4 (22, 184). Through TLR signaling, inflammatory responses including microglial activation, induction of proinflammatory cytokines, neutrophil/lymphocyte infiltration, and NF-κB activation lead to brain injury. A few studies have shown that deletion or inhibition of these TLRs improve experimental stroke outcome, whereas their overexpression exacerbates it (186, 187). However, one report showed that mice lacking TLR2 had larger infarcts and increased mortality (188) and another showed that TLR2 and TLR4 deficiency in microglia failed to ameliorate ischemic injury (189).

As mentioned previously (see Microglial/Macrophage Activation), purinergic receptors have also been studied in stroke models. Purinergic receptors bind purines, including ATP and UDP, and when activated, lead to immune cell chemotaxis and phagocytosis (190, 191). Deficiency of the P2Y12 purinergic receptor led to improved neurological outcome and reduced microglial migration (55). Of particular relevance to stroke, the P2Y12 purinergic receptor can be inhibited by the commonly used antiplatelet drug clopidogrel.

## Anti-Inflammatory Treatments

Several treatments to prevent ischemia-induced inflammation and modulate immune response have been studied in both experimental stroke and clinical trials (Table 1), which we review here.

**Table 1:** Neuroprotective agents and therapies for ischemia-induced inflammation and reperfusion injury in experimental stroke models and clinical trials.

Neuroprotective agent/therapy	Animal model	Infarct volume	Neurological outcome	Clinical trial
Minocycline (192–204)	temporary middle cerebral artery occlusion (tMCAO) rat	Reduced	Improved	Better outcome (small trial)
Integrin (205, 206)	tMCAO rabbit	Reduced	–	No efficacy
Anti-ICAM-1 antibody (207, 208)	tMCAO mice	Reduced	–	No efficacy
Anti-IL-1receptor agonist (95, 209)	tMCAO rat	Reduced	–	Better outcome (small trial)
Edaravone (210–216)	tMCAO rat	Reduced	Improved	Better outcome
Simvastatin (217)	tMCAO rat	Reduced	Improved	No efficacy (combined with tissue plasminogen activator)
Rosuvastatin (218)	tMCAO rat	Reduced	Improved	–
Pioglitazone (219)	tMCAO rat	Reduced	–	–
Sitagliptin (220)	tMCAO rat	Reduction of inflammatory mediators		–
Fingolimod (221–229)	tMCAO rat	Reduced	Improved	Better outcome
Natalizumab (230–232)	tMCAO rat	Reduced	Improved	Better outcome (small trial)
Cyclosporine A (233, 234)	tMCAO rat	Reduced	Improved	No efficacy
Glatiramer (235)	tMCAO mice	No efficacy	No efficacy	–
Intravenous immunoglobulin therapy (236)	tMCAO mice	Reduced	Improved	–
Hypothermia (237–242)	tMCAO rat	Reduced	Improved	Better outcome

### Minocycline

Minocycline is a semisynthetic tetracycline antibiotic which has pleiotropic actions (243). This agent has been shown to improve chronic inflammation in several models of neurological diseases including experimental stroke (192). Rodents subjected to focal and global cerebral ischemia showed neurological improvement with correlations to anti-inflammatory, antioxidant, and antiapoptotic effects with a somewhat wide temporal therapeutic window of at least 4 h (193–197). In fact, in one study showed that delaying minocycline treatment even 4 days post-tMCAO and continued for 4 weeks led to improved motor and cognitive functions and reduced microglial activation and improved neurogenesis (198). A major mechanism of neuroprotection is thought to be due to its ability to suppress microglial activation *via* inhibition of the MAPK p38 pathway and NLRP3 inflammasome signaling (194). Minocycline treatment was correlated with the reduction of several proinflammatory cytokines, as well as ROS and NO (197, 199). Minocycline also improved BBB viability and integrity by promoting neurovascular remodeling *via* MMP inhibition (195, 197). Monocyte chemotactic protein-induced protein1 (MCPIP1), which is expressed in monocytes following exposure to MCP-1, has also been shown to be an important mediator of minocycline-induced neuroprotection. Minocycline upregulated MCPIP1 in neurons and microglia and MCPIP1 inhibits expression of proinflammatory cytokines due to negative regulation of NF-κB signaling pathways (199).

Minocycline has also been studied in combination therapy. For example, minocycline and aspirin led to reduction of ischemic injury, brain edema, BBB disruption, and neurological dysfunction in diabetic rats subjected to tMCAO (200). MMP-2 and MMP-9 were both suppressed by treatment, as were cyclooxygenase-2 and tPA. In combination with cell based therapy, minocycline induced the pleiotropic protein Nrf2, which provided an antioxidant effect that protected transplanted neural stem cells in a stroke models (201, 202). Normobaric hyperxia plus minocycline also demonstrated synergistic effects, with greater protection together than each intervention alone.

Finally, minocycline has also been studied in a few clinical trials. In a small study, oral minocycline contributed to better outcomes as measured by lower National Institute of Health Stroke Scale [NIHSS] and modified Rankin Scale [mRS] scores, and higher Barthel Index [BI] in patients with acute ischemic stroke (203). Intravenous minocycline therapy was associated with reduced MMP-9 levels in stroke patients (204). Since minocycline is already in clinical use for other indications and has an acceptable safety profile, it could be considered as an adjunctive treatment to pharmacological thrombolysis and/or mechanical thrombectomy. Because it already in clinical use for other indications, minocycline could conceivably be given to acute IS patients.

### Fingolimod (FTY720)

FTY720 is a lipophilic immunomodulatory sphingosine-1-phosphate (S1P) analog, which is now used for treatment of multiple sclerosis and prevents T-lymphocyte egress from lymphoid organs (244). FTY720 reduces R/I to the other organs such as kidney, liver, and heart in the laboratory (221, 222). Studies in cerebral ischemia have similarly shown reduction in I/R injury and improvement in neurological function (223). One study even modeled a clinically relevant scenario of experimental thromboembolic stroke and combination treatment with fingolimod and rt-PA. This study found not only improvement in neurological outcome but also reduction in HTf (224).

FTY720 is known to act differential S1P receptors (-1 [S1P1], -3 [S1P3], -4 [S1P4], and -5 receptors [S1P5]) (225). Phosphorylated FTY720 mainly binds to lymphocyte S1P1 receptors in neuron and contributes to anti-inflammatory effect due to reduction of T-lymphocyte infiltration and proinflammatory cytokines (IFN-γ, IL-17) (226). S1P1 also plays an important role in enhancing BBB integrity (225). Once ischemic stroke occurs, S1P levels are increased and S1P-related enzymes are upregulated in the injured brain. In experimental stroke, FTY720 is associated with activation of prosurvival factors Akt and ERK (226). In addition to S1P1, FTY720 was also thought to signal through the S1P3 receptor (227). In addition to S1P receptor-related mechanisms, FTY720 may directly act to suppress microglia/macrophage activation and reduce proinflammatory cytokines, neutrophil infiltration and downregulate adhesion molecules (e.g., ICAM-1) (221, 225). FTY720 appears to have at least two temporal therapeutic windows ranging from 1 to 3 or 4 days after ischemia (suppression of microglial activation) and up to 14 days after ischemia (suppression of T-lymphocytes infiltration).

However, some studies failed to show any benefit of fingolimod. FTY720 treatment in pMCAO did not affect stroke outcome, findings that might suggest that any beneficial effect is through mechanisms exclusive to R/I (228, 229). Yet another study failed to show any benefit when fingolimod was given in combination with rt-PA, thus contradicting the results of the above referenced investigation (245).

There is some experience with fingolimod at the clinical level. In addition to its use in multiple sclerosis where it has been shown to improve disease course, FTY720 has been studied in ischemic stroke patients. Fu et al. (246) administered FTY720 6–72 h poststroke to patients with anterior cerebral circulation syndromes for 3 days. They reported that oral FTY720 could be given safely and improved neurological recovery at 90 days. FTY720 treatment also decreased microvascular permeability and circulating lymphocyte counts. While clinical experience is still limited at this point, it may be worthwhile to pursue larger trials.

### Neutrophil and Cytokine Inhibition

Antineutrophil interventions to prevent neutrophil infiltration, trafficking and/or activation have been studied at both the laboratory and clinical levels. Several reports of neutrophil depletion led to reduced infarct volume and infiltrated brain neutrophil counts (66, 71–73). Pharmacologically blocking leukocyte adhesion and migration into ischemic brain were performed in several experimental models with salutary results (102, 205) that led to the design of a few clinical studies using antibodies against adhesion molecules such as ICAM-1 and CD11/CD18 or by administering neutrophil inhibiting factor in the ASTIN trial (76, 206). However, none these studies were efficacious. In fact, the study of an anti-ICAM-1 antibody not only showed lack of benefit but also showed that this treatment made patients worse (207, 208).

Cytokine inhibiting strategies have also been studied at the clinical level. In one phase II trial, human recombinant IL-1 receptor antagonist (IL-1Ra), IL-1’s endogenous inhibitor, was studied (209). In this trial, inflammatory molecules were decreased among IL-1Ra-treated patients and these patients also had improved neurological function and smaller infarct volumes.

### Hypothermia

Hypothermia (HT) has been lauded as the prototypical neuroprotectant, and clinical studies have shown that mild to moderate cooling improves neurological outcomes following cardiac arrest and neonatal hypoxia ischemia (237). While it has yet to be shown as improving in clinical stroke outcome, several laboratory studies have shown it to be robustly protective. Particularly in tMCAO, HT appears to be robust across several laboratories and experimental models (238, 239). One mechanism underlying its beneficial effect is that therapeutic cooling seems to have remarkable effects on suppressing inflammation (240). Reduction of inflammatory molecules by HT therapy has been shown by several groups, including proinflammatory cytokines (IL-1β, TNF-α, IL-6), MMPs (MMP-9, MMP-2), and NF-κB (241). HT has been suggested as an adjunctive treatment in ischemic stroke including I/R injury (242, 247). Relevant to cotreatment with rt-PA, HT has been shown in the laboratory to extend the therapeutic time-window of other neuroprotectants, sometimes extending the therapeutic window from 2 h to over 2 months, in some cases (242). Combination therapy with HT and rt-PA at the experimental level has revealed varying results (248), but such a combination did not appear to increase HTf or increase mortality (249).

At the clinical level, the ICTuS2 study examined HT in acute stroke and included patients who were eligible for treatment with rt-PA (250). This study showed that both HT and HT plus rt-PA were both safe and feasible, but cooling increased the risk of pneumonia. In a different study, Hong et al. (247) reported in a small study that mild HT therapy (temperatures of 34.5°C) for 48 h followed by 48 h of rewarming led to reduction of HTf and brain edema and better clinical outcomes.

### Others

Edaravone is an antioxidant and ROS scavenger marketed as a neurovascular protective agent (210). Okamura et al. (211) reported that edaravone reduced infarct volume, brain edema, and HTf 24 h after stroke in tMCAO in rats. This was also correlated with reduction in inflammatory molecules, proinflammatory cytokines (IL-1β and IL-6), chemokines (fractalkine), and MMP-9 (210). At the clinical level, neurological function including NIHSS and BI was improved and several adverse events (HTf, pulmonary infection, stroke recurrence, and seizures) were also reduced by edaravone (212, 213). Edaravone is already approved for the treatment of ischemic stroke patients who present within 24 h of the onset of symptoms in Japan and other countries, but not the United States (251).

3-Hydroxy-3-methylglutaryl-coenzyme A reductase inhibitors (statins) are used for treatment of dyslipidemia and are nearly routinely prescribed for secondary prevention of stroke (252). Statins have been also studied as neuroprotectants in experimental stroke (253). Statins have been correlated with upregulation of eNOS, inhibition of ROS-induced lipid peroxidation, reduction of superoxide as well as anti-inflammatory effects (254). A variety of statins have been shown to reduce infarct volume and improve neurological deficit in experimental stroke (217). Rosuvastatin was also shown to reduce R/I by inhibiting oxidative stress and inflammatory responses such as reducing superoxide and NOX, inhibiting microglial activation, and downregulating of inflammatory molecules (NF-κB, iNOS) (218). In clinical studies, Simvastatin has been shown to reduce subsequent strokes and improve neurological function, but efficacy has not been shown in combination with tPA (255, 256).

Several antidiabetic drugs have been shown to have a neuroprotective effect. Kumari et al. (257) reported that treatment with peroxisome proliferator-activated receptor γ (PPARγ) agonist attenuated infarct volume and reduced inflammatory responses in experimental stroke. Generally, PPARγ activation in monocyte-derived macrophage has been thought to influence macrophage polarization (258). It also affects ischemia-induced inflammation and HTf (257). Hence, PPARγ agonists act not only to decrease serum glucose levels but also to decrease inflammation. Further, the PPARγ agonist pioglitazone not only protected from tMCAO but also was associated with antioxidant and antiapoptotic effects in addition to anti-inflammatory effects in diabetic rats subjected to tMCAO (219). PPARγ agonists have pleiotropic effects against ischemia/R/I, as do dipeptidyl peptidase-4 inhibitors. In a recent study of diabetic rats subjected to experimental stroke, sitagliptin administration give before I/R was shown to suppress NF-κB signaling (220). In parallel, it was also shown to be anti-inflammatory, antioxidant, and antiapoptotic.

Other immunosuppressive agents used for inflammatory diseases have been also evaluated for anti-inflammatory and neuroprotective effects in ischemic stroke. Natalizumab, a humanized monoclonal antibody to CD49d, has been used for treatment of multiple sclerosis. Similar approaches have also been studied in acute ischemic stroke (230). Using a related antibody, Relton et al. (231) treated rats subjected to tMCAO with antibody to the CD49d into and showed that it reduced infarct volume and improved neurological deficits. Treatment also led to reduction of lymphocyte infiltration and VCAM-1 upregulation. However, other studies did not show efficacy using this approach. The ACTION clinical trial explored safety and efficacy of natalizumab in stroke (232). Natalizumab did not affect infarct volume or neurological outcomes (mRS and BI) at 90 days; thus, it seems unlikely that natalizumab might be further developed for this indication. There are also side effects occasionally seen in patients with multiple sclerosis treated with this agent. Glatiramer is another compound used in the treatment of MS (259). Its anti-inflammatory mechanism is still unclear, but it appears to inhibit Th1 proinflammatory cytokines, while inducing Th2/T_reg_ activation. Kraft et al. (235) studied glatiramer in an experimental stroke model. While microglial activation and proinflammatory cytokine generation were suppressed, it failed to reduce ischemic lesion volume and neurological deficits. Cyclosporine A, a calcineurin inhibitor used for variety of immune diseases, in stroke models led to the reduction of infarct size and inflammation through the suppression of cytokines, T cell activation, and ROS production, but failed to show efficacy in clinical trials (233, 234). Finally, intravenous immunoglobulin (IVIG) therapy has been shown to have neuroprotective and anti-inflammatory effects in ischemic stroke models by modulating the TLRs and MARK pathways (236). While IVIG is used routinely at the clinical level for other indications, it has yet to be studied in stroke patients.

## Anti-Inflammatory Treatment Combined with Acute Revascularization

Recent studies have demonstrated the efficacy of anti-inflammatory treatments in combination with rt-PA in experimental stroke models. A few clinical studies have similarly focused on combined endovascular treatment (EVT) with anti-inflammatory agents (Table 2).

**Table 2:** Combined therapy with acute recanalization against ischemia-induced inflammation and reperfusion injury in experimental stroke models and clinical trials.

Neuroprotective agent/therapy	Animal model	Infarct volume	Neurological outcome	Hemorrhagic transformation	Clinical trial
Edaravon (214–216)	–	–	–	–	Better outcome (with thrombolysis and thrombectomy)
Minocycline (260–262)	temporary middle cerebral artery occlusion (tMCAO) rat	Reduced	Improved	Reduced	Only safety (with thrombolysis)
Progranulin (263)	tMCAO rat	Reduced	Improved	Reduced	–
Granulocyte-colony stimulating factor (264)	tMCAO rat	Reduced	Improved	Reduced	–
Fingolimod (265)	tMCAO mice	–	Not improved	–	Better outcome (with thrombolysis, small trial)
Hypothermia (247–250, 266)	–	–	–	–	Only safety (with thrombolysis and thrombectomy)

### Pharmacological Thrombolysis

The benefit of rt-PA therapy has been established in clinical trials (1, 2) but its therapeutic time-window is limited to 3–4.5 h from symptoms onset (267). Thus, patients who can receive rt-PA therapy are still limited (3.4–5.2% of all patients with acute ischemic stroke) and this is largely due to the risk of R/I, particularly the complication of cerebral hemorrhage which can worsen outcome and increase mortality (268). tPA is a serine protease and can thus promote ECM damage which can lead to HTf (269). Hence, combined therapy with agents that suppress inflammatory molecules, thus preventing BBB disruption and downstream effects such as edema and hemorrhage seem viable approaches.

As described in section “Others,” edaravone is generally used for acute IS treatment. Combination treatment with edaravone and rt-PA is also already used at the clinical level in Japan. The PROTECT4.5 trial compared rt-PA with rt-PA plus edaravone in acute ischemic stroke patients within 4.5 h after symptom onset. This study showed that combination therapy accelerated recanalization and reduced the incidence of cerebral hemorrhage (214). The YAMATO study was then carried out to determine whether early (vs. late) edaravone infusion promoted early recanalization and reduced complications, but failed to show any difference in outcome with either paradigm (215).

Combination treatment with rt-PA and hypothermia has been also studied both in the lab and at the clinical level, as described in section “Hypothermia.” Tang et al. (249) reported that rt-PA plus HT reduced brain hemorrhage, BBB disruption in mice exposed to tMCAO. They also showed that HT reduced endogeneous tPA expression. In a clinical trial [Reperfusion and Cooling in Cerebral Acute Ischemia (ReCCLAIM)], Horn et al. (270) showed that HT could be carried out safely after tPA therapy in patients with large infarcts. This study also suggested that this approach could reduce R/I, as outcomes were improved compared to stroke patients who did not received HT.

While minocycline did not affect rt-PA’s fibrinolytic activity and clot lysis when given in combination in an experimental stroke model (260). Fan et al. (261) reported that the combination therapy of rt-PA with minocycline was effective in hyperglycemia/type 1 diabetes rats subjected to focal cerebral ischemia. In this study, they showed that neutrophil infiltration, microglia activation, MMP-9 activity and tight junction protein claudin-5 degradation was reduced with combination therapy, compared to rt-PA alone. Minocycline is expected to reduce acute inflammatory cascades stimulated by thrombolysis. Kohler et al. (262) demonstrated the safety of this combination therapy in a pilot clinical study; however, efficacy in this small study could not be demonstrated, thus, emphasizing the need for future a larger clinical trial.

Progranulin (PGRN), a growth factor induced in the brain *via* estrogen, may prove to be a novel adjunctive treatment to rt-PA (271.) PGRN has been also reported to inhibit inflammatory reactions in stroke models. In tMCAO, PGRN increases especially within microglia and vascular endothelial cells 24–72 h after reperfusion (263). PGRN is thought to contribute anti-inflammatory and vaso-protective properties. PGRN knockout mice subjected to stroke had decreasing IL-10 levels with increasing VEGF levels compared to wildtype (263). Combination therapy with rt-PA and PGRN was beneficial in a stroke model where it not only reduced infarct size, it also reduced complications of R/I such as brain edema and hemorrhage, while improving neurological functions.

Granulocyte-colony stimulating factor (G-CSF) is thought to provide neuroprotection due to its anti-inflammatory effect (272). dela Peña et al. (264) reported that combined therapy with rt-PA and G-CSF reduced HTf and improved of neurological function 24 h after tMCAO rats, compared with rats receiving only rt-PA. G-CSF may also be expected to enhance endothelial cell survival and reduce their activation; thus, this combination may prevent BBB disruption and reduce HTf. Although G-CSF therapy in ischemic stroke patients has not been shown to be efficacious, its safety was proven for use in other diseases (273, 274). Future studies should focus on combination therapy of G-CSF and rt-PA.

Fingolimod has also been studied in combination with rt-PA. A pilot study showed that this combination was safe and had a possibility to reduced infarct volume, hemorrhage, and neurological deficit in acute stroke patients (265). In addition to fingolimod’s anti-inflammatory effects, it combination with rt-PA may also prevent BBB disruption, thus reducing hemorrhage risk.

### Endovascular Treatment

Recent randomized controlled trials have shown the efficacy of EVT within 4.5–12 h after the onset of ischemic stroke (3). The stent retriever appears to be particularly effective and safer than earlier devices. However, the rate of good clinical outcomes by EVT is still limited and adjunctive neuroprotective/anti-inflammatory therapy may be desirable in order to reduce complications, likely results of R/I. Edaravone is already used in combination with EVT in Japan. The RESCUE Japan Registry analyzed the efficacy of edaravone in patients with acute cerebral large vessel occlusion (216). Edaravone was found to be more effective for the patients treated with IV rt-PA than EVT, although the reasons for difference were unclear.

Hypothermia has been used together with EVT. Chen et al. (266) reported that selective brain cooling by intra-arterial infusion of cold saline during endovascular recanalization was feasible and safe; however, this study did not assess efficacy.

### Ongoing Trials

There are now several clinical trials evaluating the safety and efficacy of the combined therapy against ischemia-induced inflammation and R/I (Table 3). Activated protein C (APC) is an endogenous serine protease which has been detected in both neurons and endothelial cells (275). APC can suppress microglial activation and leukocyte infiltration *via* the suppression of NF-κB and proinflammatory cytokines and adhesion molecules. APC also activates a family of G-protein coupled protease-activated receptor1 and prevents BBB disruption (276). Thus, APC may be a novel adjunctive therapy to EVT because of its anti-inflammatory and BBB stabilizing properties. The “Safety Evaluation of 3K3A-APC in Ischemic Stroke (RHAPSODY)” trial (NCT02222714) which studies the safety and efficacy of adjunctive APC with rt-PA, mechanical thrombectomy, or both is ongoing (276).

**Table 3:** Recent on going clinical trials of combined therapy with acute recanalization against ischemia-induced inflammation and reperfusion injury.

Trials (neuroprotective agent/therapy)	Study design	Patients number	Treatments	End point
RHAPSODY (3K3A-APC)	Randomized, double-blined study	*n* = 110 (age 18–90 years and NIHSS ≥ 5)	5 doses (120, 240, 360, 540 µg, and placebo) with rt-PA and/or EVT	Safety, pharmacokinetics, and preliminary efficacy of multiple ascending doses
STARS07 (Simvastatin)	Double-blind, randomized, controlled study	*n* = 340 (age over 18 years)	Combination of simvastatin (started within 12 h from onset) plus rt-PA	Safety and efficacy
SEATIS (Atorvastatin)	Randomized, open label study	*n* = 256 (age over 18 years and mRS before onset less than 2)	2 different atorvastatin doses (80 and 20 mg) within 24 h after intravenous rt-PA for at least 2 weeks	Safety and efficacy (NIHSS, mRS, mortality, hemorrhagic complications, and recurrence)
ReCCLAIM-II (hypothermia)	Randomized, open label study	*n* = 85 (age ≥18, ≤79 years, ASPECTS 5–10, NIHSS 14–29, mRS of 2 before onset)	Reducing body temperture (mild hypothermia of 33°) with acute revascularization (rt-PA and EVT), compared to normothermia group	Efficacy (hemorrhage conversion, hyperintense acute reperfusion marker, NIHSS, and mRS), safety (adverse events)

*rt-PA, recombinant tissue plasminogen activator; EVT, endovascular treatment; NIHSS, National Institute of Health Stroke Scale; mRS, modified Rankin Scale; ASPECTS, Alberta Stroke Programme Early CT Score*.

In preclinical studies, statins have been shown to have neuroprotective properties through their ability to upregulate eNOS (277). Now, there are a few clinical trials to evaluate their efficacy in combination with reperfusion strategies. “Stroke Treatment with Acute Reperfusion and Simvastatin (STARS07)” trial and “The Safety and Efficacy Study of High Dose Atorvastatin after Thrombolytic Treatment in Acute Ischemic Stroke (SEATIS)” trial (NCT02452502) are undergoing to evaluate the efficacy (mortality, recurrence, ischemic lesion volume, HTf, NIHSS, BI, and mRS) and safety.

There is also another clinical trial in progress to study neuroprotective therapy in association with acute recanalization against ischemia-induced inflammation and R/I. The “ReCCLAIM-II” trial (NCT02411877) compares mild hypothermia to normothermia in patients treated with rt-PA and/or EVT and evaluates its effect on neurological efficacy, hemorrhagic conversion, and imaging markers of R/I.

## Conclusion

Inflammation and R/I following ischemic stroke can worsen outcome, and this issue becomes particularly important now that there are two broad categories of effective acute revascularization treatments. In this review, we focused mainly on inflammatory mechanisms and targets to prevent R/I. In reviewing the literature, many experimental studies did not necessarily focus on reperfusion as a target, but inflammation modulating interventions for neuroprotection. Similarly, the many failed clinical stroke studies of neuroprotection were carried out before these treatments were approved. There are certainly differences between clinical trials and experimental models regarding the mechanisms of reperfusion (or lack of reperfusion in early clinical studies) and the alignment or misalignment of treatment timing. However, recent progress in acute revascularization at the clinical level open the possibility of revisiting neuroprotectants to further improve outcomes in a synergistic manner. As we are now in the age of restoring CBF, it seems worthwhile to address these neuroprotective treatments once again. Perhaps combination therapies with anti-inflammatory treatments given to coincide with the onset of reperfusion should be the focus of future laboratory and clinical studies with purpose of determining whether adjunctive treatment may reduce complications, further improve neurological outcomes and even determine whether the time windows for intervention could be lengthened.

## Author Contributions

This review article was written by AM, including a thorough review of the literature and preparation of figures and table. MY developed the outline, made revisions, and approved the final version.

## Conflict of Interest Statement

The authors declare that the research was conducted in the absence of any commercial or financial relationships that could be construed as a potential conflict of interest.
